# Overexpression of E6/E7 mRNA HPV Is a Prognostic Biomarker for Residual Disease Progression in Women Undergoing LEEP for Cervical Intraepithelial Neoplasia 3

**DOI:** 10.3390/cancers15174203

**Published:** 2023-08-22

**Authors:** Maria Teresa Bruno, Giulia Bonanno, Francesco Sgalambro, Antonino Cavallaro, Sara Boemi

**Affiliations:** 1 Department of General Surgical and Medical-Surgery Specialities, University of Catania, 95124 Catania, Italy; 2 Multidisciplinary Research Center in Papillomavirus Pathology, University of Catania, 95124 Catania, Italy; saraboemi@live.it; 3 Department of Obstetrics and Gynaecology, Azienda di Rilievo Nazionale e di Alta Specializzazione (ARNAS) Garibaldi Nesima, 95124 Catania, Italy; giulyibonanno.gb@gmail.com; 4 Obstetrics and Gynecology Unit, University Hospital “G. Rodolico”, 95100 Catania, Italy; f.sgalambro@policlinico.unict.it (F.S.); ninocavallaro@tin.it (A.C.)

**Keywords:** LEEP, CIN3, HPV persistence, positive margin, residual disease, E6/E7 mRNA HPV

## Abstract

**Simple Summary:**

Long-term population-based studies have demonstrated that the risk of cervical cancer after conization for CIN3 treatment persists for at least 25 years, underscoring the need for careful follow ups and the importance of detecting residual disease after LEEP. Data from the literature show that positive-margin and post-treatment HPV persistence are predictors of residual disease. The management of these cases determines the use of a second LEEP or an accurate follow up, but the risk of overtreatment or of not treating an occult carcinoma exists. Our goal was to discover an efficient method to select patients requiring a second LEEP from those requiring a follow up (FU) only through the use of E6/E7 HPV mRNA search. This prognostic marker allowed us to identify women with residual disease (CIN2+) and treat them with a second LEEP. At the same time, it helped us identify E6/E7-mRNA-negative women as patients at low risk of progression, potentially avoiding further treatment and subjecting them to a follow up only.

**Abstract:**

The risk of overtreatment or not treating an occult carcinoma exists in women at risk of residual disease after a LEEP excision for CIN3. Our goal was to discover an efficient method to select patients requiring a second LEEP from those requiring a FU only through an mRNA-detection test. In a population of 686 women undergoing a LEEP excision for CIN 3, we selected 285 women at risk of residual disease and subjected them to a search for E6/E7 mRNA HPV. The women with negative mRNA were subjected to a follow up, while the women with positive mRNA were subjected to a second LEEP. The histological examination of the second cone revealed 120 (85.7%) cases of residual disease in the mRNA-positive women: 40 cases of CIN2, 51 cases of CIN3, 11 cases of squamous microinvasive carcinoma, 7 cases of squamous carcinoma, 9 cases of AIS (adenocarcinoma in situ) and 2 cases of adenocarcinoma. Among the mRNA-negative women undergoing a follow up, there were only five cases of residual disease. During the follow-up period of about 6 years, we witnessed the regression of the residual disease and the elimination of the virus, just as predicted by the negative result of the mRNA test. Testing patients for E6/E7 mRNA allowed us to identify women with residual disease (CIN2+) and treat them appropriately.

## 1. Introduction

Papillomavirus infection is the most frequent sexually transmitted infection in the world [1]. Its incidence is higher in the younger segment of the population, adolescents and young women at their sexual onset. In 80% of cases, within 18–24 months, there is clearance of the virus [2,3]; however, in cases of the viral persistence of high-risk strains, there is an increased risk of preneoplastic lesions of the cervix which, if left untreated, can lead to the onset of cervical cancer over the years [4,5]. CIN3, a true precursor of cervical cancer, has to be treated with excisional treatments that can be performed with a cold blade or by LEEP.

The removal of the cervical lesion as well as the entire squamocolumnar junction (SCJ) is the principle goal of LEEP (a large loop excision of the transformation zone). This procedure is considered the optimal treatment method for CIN3. Meta-analyses have shown that cone depth is associated with the risk of preterm birth; however, a more conservative treatment of CIN3 could potentially leave residual disease and infected tissue with the risk of progression causing a post-LEEP “positive margin” and “persistence of HPV infection”, respectively, both identified by the literature as risk factors for residual disease/lesion recurrence [6,7].

Long-term population studies have shown that the risk of cervical cancer after conization for the treatment of CIN3 persists for at least 25 years, underlining the need for a careful follow up and the importance of detecting the presence of residual disease, which is the diagnosis of CIN2+ at the first evaluation after conization [8].

The “positive margin” was proposed as an accurate predictor for residual disease after conization. Generally, the positive margin is managed by a regular follow up or a second LEEP. Data from the literature report that almost half of the cases with a positive margin do not develop recurrent or residual disease, and that even patients with a negative margin may have residual disease [9]. The risk of overtreatment or not treating an occult carcinoma exists. Furthermore, a second LEEP increases the negative effects of a cervical excision on the risk of preterm labor, adverse neonatal outcomes and the sexual health of young women [10,11,12]. Therefore, the identification of patients to undergo a second LEEP or follow up cannot be entrusted solely to the nature of the resection margins.

A meta-analysis by Arbyn et al. showed that HPV testing was more accurate than margin status in predicting residual disease, with an increased sensitivity (91% vs. 56%) and equivalent specificity (84%) [13].

HPV testing has helped improve the diagnosis of post-LEEP residual disease; however, it shows a reduced specificity that limits the diagnostic accuracy [13]. Currently, the guidelines recommend the use of an HPV test and a pap test (cotest) in the follow up of the woman after conization for CIN3 while considering the limits of low specificity that the HPV test shows.

The progression to cancer does not occur due to the presence of the virus but to the integration of the E6 and E7 genes and the overexpression of their transcripts; therefore, the demonstration of HPV E6/E7 transcripts in cervical samples may be more specific than HPV DNA testing alone.

In 2010, we introduced into our clinical practice the use of the E6/E7 HPV mRNA test for the management of HPV-positive women. We also started using the test in the follow up of women treated with LEEP for CIN3. The high sensitivity and specificity make the mRNA test a valid diagnostic and prognostic marker [14,15] that has allowed us to identify cases of residual disease to be followed up on, avoiding cases of overtreatment and, at the same time, cases to be subjected to a second LEEP without running the risk of not treating occult cancer. In this study, we collected retrospective data from women at risk of residual disease (women with a positive margin and/or persistent HPV) and subjected them to a protocol based on the use of the E6/E7 mRNA HPV test, subjecting positive women to a second LEEP and negative women to a follow up.

## 2. Materials and Methods

### 2.1. Study Population and Design

#### 2.1.1. Baseline Population

We performed a multicenter retrospective study; data from women who underwent cervical conization (LEEP) for CIN3 from January 2012 to December 2018 were collected into a dedicated database. We considered women who underwent LEEP for CIN3; had a margin status description of their histological examination; were positive for one or more of the following genotypes: 16, 18, 31, 33 and 45; and had performed an HPV test before and after the LEEP. All women were included in this study if they had undergone a second LEEP because of positive mRNA or were subject to a follow up because of negative mRNA, or if they had completed a 6-year follow up.

Women who underwent LEEP for a histology other than CIN3, did not have follow-up data, were younger than 18 years old, had a positive pregnancy test or had a history of cervical cancer were excluded.

All the women underwent an electrosurgical conical excision (conization) and/or diathermic loop surgical treatment (LEEP).

Residual disease is the diagnosis of CIN2+ at the first post conization assessment; low-grade cervical lesions (LSIL/CIN1) were not considered as residual disease. The identification of the same type of HPV before and after the LEEP was considered HPV persistence.

#### 2.1.2. Follow-Up Procedure

In the presence of positive margins, the first follow up is carried out three months after LEEP.

The follow-up protocol included a pap test/repeated HPV test followed by a colposcopy every 6 months for two years and annually thereafter; if three subsequent cotests were negative, attendance occurred every three years.

According to our protocol, women with positive margins and/or HPV persistence (285 women) were tested for E6/E7 HPV mRNA, women who tested positive underwent a second LEEP and negative women were followed up on. A histological examination of the second cone was reviewed by two pathologists.

Written informed consent regarding the use of data for scientific purposes was obtained from all the participating patients.

The University Hospital’s ethics committee waived the requirement of ethical approval and informed consent because the study used previously archived data.

### 2.2. LEEP Technique

LEEP was performed with colposcopic guidance under local anesthesia in the clinic by experienced personnel; loops that were 20 mm wide and 12, 15 or 20 mm deep were used depending on the characteristics of the lesion and the conformity of the cervix. The resection margins were kept 2–3 mm beyond the lesion, and the completeness of the lesion removal was verified colposcopically. A histological examination of the cervical cone established the definitive histological diagnosis and evaluated the involvement of the cone margins. The margins of the cone were reported as positive if the distance between CIN2+ and the resection surface was <1 mm.

### 2.3. HPV Testing and Genotyping

After cytological sampling for HPV DNA, the samples were sent to the laboratory for DNA extraction [16] and viral DNA genotyping by genetic amplification followed by hybridization with genotype-specific probes capable of identifying most of the HPVgenotypes of the genital region (18 high-risk HPV genotypes (16, 18, 26, 31, 33, 35,39, 45, 51, 52, 53, 56, 58, 59, 66, 68, 73 and 82), 7 low risk (6, 11, 40, 43, 44, 54 and 70) and 3 undefined risk (69, 71 and 74)). The commercial method used was the MAG NucliSenseasy system (bioMérieux SA, Marcy l’Etoile, France). The DNA amplification technique was previously described in reference [17].

### 2.4. Detection of E6/E7 mRNA HPV

HPV E6/E7 mRNA amplification and detection were performed with the PreTect HPV-Proofer real-time multiplex NASBA test, using a primer/probe PCR for HPV types 16, 18, 31, 33 and 45. The total DNA/RNA was extracted by using an Easy-Mag automatic extractor (bioMerieux S. [18] A., Marcy l’Etoile, France) according to the manufacturer’s instructions. The oncogenic E6/E7 transcripts of the five high-risk HPV types 16, 18, 31, 33 and 45 were detected by the NucliSense EasyQ HPV kit (bioMerieux S.A., Marcy l’Etoile, France) that uses nucleic-acid-sequence-based amplification technology (NASBA) to amplify the viral target. Six different molecular beacons are used to identify and amplify the corresponding five HPV types and the U1A gene. Two different fluorophores, 6-carboxyfluorescein (6-FAM) for HPV 16, 31 and 33 and 6-carboxy-X-rhodamine (6-ROX) for U1A, HPV 18 and 45 allow for simultaneous duplex amplification. Dedicated software reveals the presence or absence of the viral target. The PreTect HPV-Proofer real-time multiplex NASBA test was performed as suggested by the manufacturer (NorChip AS, Klokkarstua, Norway). Briefly, three premixes were made by reconstituting the reagent sphere, containing nucleotides, dithiothreitol and MgCl2, in a diluent of the reagent sphere (Tris-HCl, 45% dimethyl sulfoxide). Then, the primer–molecular beacon mixture U1 ribonucleoprotein A (U1A)-HPV-16, HPV-33-HPV-45 or HPV-18-HPV-31 specific for small nuclear proteins was added together with a KCl stock solution. Ten microliters of this premix were distributed into each well in a reaction plate, followed by the addition of RNA and 4 min of incubation at 65 °C (to destabilize the secondary RNA structures) and 4 min of incubation at 41 °C. The reaction was initiated by adding enzymes (avian myeloblastosis virus reverse transcriptase, RNase H and RNA polymerase T7) and measured in real time by using a Lambda FL 600 fluorescence reader (Bio-Tek, Winooski, VT, USA) at 41 °C for 150 min. The total volume of the reaction was 20 μL. A newly developed software package (PreTect analysis software: NorChipCOME, Klokkarstua, Norway) was used. The excitation (nm) filters for 6-carboxyfluorescein and Texas Red were 485/20 and 590/20, respectively, and the λ (nm) emission filters were, respectively, 530/25 and 645/40. The RNA isolated from CaSki cells was used as a positive control for HPV-16. Artificial and standardized oligonucleotides corresponding to the viral sequence were used as positive controls for HPV types 18, 31, 33 and 45. As a performance check, to avoid false negative results due to RNA degradation, we used a set of primers and a probe directed against human U1A mRNA. Negative controls consisting of all reagents except RNA were included in each run.

## 3. Results

**Figure 1:**
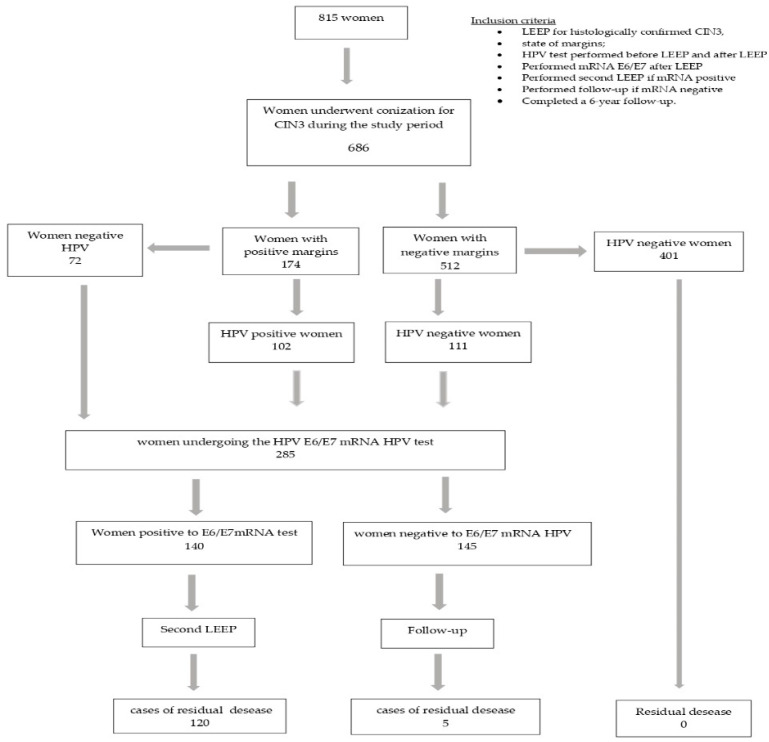
Flow-chart of study population.

Of the 815 patients who underwent LEEP, only 686 met our requirements: 37 cases had a histological diagnosis of CIN1; 52 asked to undergo a simple hysterectomy; and in 40 cases, the margin assessment was ambiguous. After the primary LEEP, 174/686 (25.4%) women had positive margins and 512/686 (74.6%) had negative margins; 213 (31%) women had hr-HPV persistence and 473 (69%) were virus free (Figure 1).

We tested 285 women at risk for residual disease (HPV-positive and/or margin-positive women) for mRNA HPV: 174 women had a positive margin, 111 women had a negative margin with viral persistence and 72 women had a positive margin but were negative for HPV infection (Table 1).

The 140 women who tested positive for E6/E7 HPV mRNA underwent a second LEEP excision, and the 145 negative women were followed up on.

The residual disease among the 140 patients who underwent a second LEEP was 85.7%, and a histological examination of the second cone revealed 120 cases of residual disease: 40 cases of CIN2, 51 cases of CIN3, 11 cases of squamous microinvasive carcinoma, 7 cases of squamous carcinoma, 9 cases of AIS (adenocarcinoma in situ) and 2 cases of adenocarcinoma (Table 2).

Among the 145 women with a negative mRNA test result and subjected to a follow up, we had only five cases of residual disease: three cases of CIN3 and two cases of CIN2. Table 3 shows the cases of residual disease (RD) according to the mRNA results.

Based on the state of the resection margins, we had 80 (46%) cases of residual disease in women with positive margins and 45 (40.5%) cases in women with negative margins, OR: 1.25 (CI 95% 0.8–2.0), *p* = 0.001 (Table 4).

Considering the viral status, in our study, the 6-month persistence rate was 31%; 110 (51.6%) women with a persistent high-risk genotype had residual disease, and only 15 (3.17%) HPV-negative women with positive margins had residual disease, OR = 4.06 (CI 95% 2.2–7.6), *p* = 0.001. None of the HPV-negative and margin-negative women had residual disease. Genotype 16 had the highest persistence rate. As in other studies [5,7,15], HPV16 was the most frequently detected genotype in our study. The second most common type was HPV31, followed by HPV33 and HPV18.

Positive resection margins and viral persistence, particularly of genotype 16, after the primary LEEP were significant predictors of residual disease (Table 3). The sensitivity, specificity, VPP and VPN for residual disease prediction was 64% (95%CI 58.1–69.5%), 41.3% (95%CI 95% 35.5–47.2%), 46% (95%CI 95% 40–52%) and 59.5% (95%CI 53.5–65.2%), with positive margin, respectively, and 88% (95%CI 83.5–91.4%), 35,6% (95%CI 30.1–41.5%), 52% (95%CI 46–57.6%) and 79.2% (95%CI 74–83.6%), respectively, for those who took an HPV test. (Table 5)

## 4. Discussion

We had 125 (18.2%) cases of residual disease. In the literature, data on recurrence or residual disease are very variable as many studies do not clarify the definition of recurrence and residual disease and often do not distinguish between the term “relapse” and “residual disease”, using the generic term recurrent disease, leading to results that can have a vague interpretation. In our study, a woman with residual disease after LEEP still has a histological diagnosis of CIN2+, while recurrence entailed at least one negative examination between the primary LEEP and diagnosis of recurrence (CIN2+). In addition, in some studies, positive margins are defined as a histological diagnosis of CIN along with the margin of the LEEP sample, regardless of the CIN grade.

Our results indicate that patient identification for a second LEEP based only on resection margin status would have resulted in the overtreatment of many young women and the insufficient treatment of a significant proportion of women; approximately 54% of the women in our series (94/174) with positive margins had no residual disease (CIN2+), while 45 (40.5%) women with negative margins had residual disease, which is compatible with the literature data that reported that 23–31% of patients with negative margins may have residual disease after LEEP and that 37–60% of patients with positive margins may have no residual disease [9,19]. A positive margin does not always correspond to a residual disease on the cervix as the thermal and coagulative effect produced by LEEP can have an ablative effect on the lesion; moreover, the residual disease can be found on the endocervical margin and not be diagnosed, or the lesion can regress spontaneously following activation of the immune system [20,21]. Conversely, even patients with a negative margin may have residual disease from multiple primitive lesions of the cervix, which cannot be predicted from the incisional margin, especially if the lesion is glandular in nature [22]. In our study, approximately 6.4% (nine cases) of E6/E7 HPV mRNA-positive patients who underwent a second LEEP had Skip lesions, associated with a high risk of residual lesions (Table 2). Therefore, a negative margin cannot completely exclude the diagnosis of residual disease. LEEP performed for squamous cell abnormalities should be carefully evaluated for glandular lesions.

An occult microinvasive and invasive disease was observed in 24.2% of the residual disease cases, with 9 cases of invasive carcinoma and 20 cases of microinvasive carcinoma, all in women with a positive margin. The diagnosis of cervical cancer in women with positive margins varied between studies [23,24]; some authors have underlined an incidence between 0.9–9.6% [25]. In our study, the incidence of cervical cancer in women with a positive margin was 11.2%, with seven cases of invasive squamous cell carcinoma and two cases of invasive adenocarcinoma. Our higher rate is related to the selection of only patients with CIN3. In our study, all occult invasive lesions had positive margins, whereas 54.5% (94 women) of the women with positive margins had no residual disease, so a second LEEP would have been an overtreatment. In addition, 40.5% of women with a negative margin but with residual disease were at risk of not being treated due to their resection margin status. The sensitivity, specificity, PPV and VPN of the positive margin in predicting residual disease was 64%, 41.3%, 46% and 59.5%, respectively. These data confirm the data of the literature [6].

Our data also support that a persistent HPV status after conization predicts residual disease more accurately than the margin status.

We had a viral persistence at 6 months of 31%; other authors reported lower 6-month HPV persistence rates, ranging from 14.3% to 21.5% [26]. Our higher persistence rate is related to the selection of only patients with CIN3 and the fact that all patients in our study were positive for the five most oncogenic genotypes (16, 18, 31, 33, 45). As in another study [27], HPV16 was the most commonly detected genotype in our study. In our data, the postoperative HPV genotypes were almost the same as the preoperative ones. The meta-analysis by Arbyn et al. concluded that a positive hr HPV DNA test result predicts treatment failure more accurately than positive resection margins [28]. The potential role of HPV testing in predicting residual disease was further confirmed in our case series; 51.6% of HPV-positive women after the primary LEEP had residual disease, and only 3.5% were HPV negative, OR = 4.06 (95%CI 2.16–7.61), *p* = 0.001. No patients with negative resection margins and negative HPV tests were shown to have residual disease. The sensitivity, specificity, PPV and NPV of the HPV test were higher than those for the resection margin, as confirmed by other authors [29]. The HPV test has certainly added a high sensitivity; however, despite this, some limitations have also been highlighted, especially with regard to low specificity [30]. The test does not distinguish between regressive and progressive lesions; consequently, it can lead to overdiagnosis (101 HPV positive women (47.4%) had no residual disease), resulting in the overtreatment of these patients.

We used the E6/E7 mRNA search as a marker, subjecting only positive cases to a second LEEP. The mRNA test is used in clinical practice to study the ability of CIN progression or regression in HPV-positive women [15]. Compared to DNA-based tests that only indicate the presence or absence of viruses, the detection of E6/E7 mRNA HPV provides more information on viral activity. Recent evidence has also shown that the detection of mRNA transcripts of HPV E6/E7 may provide greater specificity for the detection of high-grade cervical lesions since the oncogenic potential of an HPV infection depends on the overexpression of these two transcripts [31]

With a high specificity and NPV, the HPV E6/E7 mRNA test compensates for the low specificity of DNA tests for the clinical detection of high-grade cervical lesions [32].

In our study, it showed an excellent sensitivity (86%), excellent specificity (97%), PPV of 96% and NPV of 87.5%. These values make mRNA an excellent marker of progression, but also of regression in its negative form, reducing cases of overdiagnosis and consequent overtreatment. The positivity of the test identified the women to be subjected to a second LEEP, and the histological examination of the second cone showed 85.7% (120/140) of the cases of residual disease. Moreover, in 15 cases, the integrated viral genome was no longer visible to the DNA test but was detected by the E6/E7 mRNA test [33,34]; this result, which would have escaped the HPV test, strengthens the diagnostic capacity of the mRNA test and also acts as a risk marker for lesion progression.

Among the mRNA-negative women undergoing a follow up, there were only five cases of residual disease. During the follow-up period of about 6 years, we witnessed the regression of CIN2+ (three cases of CIN2 and two CIN3) and the clearance of the virus, just as predicted by the negative result of the mRNA test. Therefore, our results show that even in cases where residual disease (CIN2+) is present, it is not necessary to resort to a second LEEP but to be guided by markers capable of indicating the risk of the progression (mRNA+) or regression (mRNA−) of the lesion. In addition, women with negative HPV E6/E7 mRNA may increase the follow-up interval, reducing the rate of colposcopy and biopsy [35].

This study is the first of our knowledge to evaluate the usefulness of HPV E6/E7 mRNA testing to identify women with residual disease to be subjected to a second LEEP.

In our study, a woman with a positive mRNA test has a risk of 168 (95%CI; 61.20–461.20), *p* = 0.001, of having a residual disease that progresses; the risk drops to 4.06 if the woman is hr HPV positive and to 1.25 if the woman has positive margins.

This study has several strengths, including a relatively large number of patients and a long-term follow-up (6 years), the multicenter design and the homogeneity of the patients included.

The inclusion of a second histopathologic review of the second cone to confirm the diagnosis of residual disease further strengthens the validity of the results.

An intrinsic limitation is the design of the retrospective study, as it generates a great deal of incomplete data; and, the NASBA technique used to search for E6/E7 mRNA referred only to five genotypes: 16, 18, 31, 33 and 45, which are the most oncogenes [36,37]. Searching for only 5 genotypes increases the specificity of the test, compared to the other two techniques on the market, Aptima and Quantivirus, capable of searching for 14 genotypes. Furthermore, we only analyzed the difference between positive and negative margins but did not further subdivide them into endocervical margins or ectocervical margins. Further prospective studies on large samples are needed to confirm our findings.

## 5. Conclusions

The literature data show that margin status and post-treatment HPV testing are predictors of residual disease. To this now-accepted evidence, our study adds the usefulness of HPV mRNA testing as the most accurate predictive marker of residual disease, and we show that secondary treatments such as a second LEEP or hysterectomy would not be needed in a large number of cases. Testing patients for E6/E7 mRNA allowed us to identify women with residual disease (CIN2+) and treat them appropriately. At the same time, it helped us identify negative E6/E7 mRNA women at low risk of progression, potentially avoiding further treatment and subjecting them only to a follow up.

## Figures and Tables

**Table 1:** The 285 women undergoing E6/E7 HPV mRNA.

		*n*	E6/E7 mRNA+	E6/E7mRNA−
Positive margins	HPV+	102	75	27
Negative margins	HPV+	111	50	61
Positive margins	HPV−	72	15	57
Total		285	140	145

**Table 2:** Residual disease in 285 women subject to E6/E7 mRNA test according to the histologic results.

		CIN2	CIN3	Microinvasive Carcinoma	Invasive Carcinoma	AIS	AdenoK	Overall
Positive-mRNA women subjet to second LEEP	Positive margins HPV positive	17	31	8	7	0	2	65
	Positive margins HPV negative	3	0	3	0	9		15
	Negative margins HPV positive	20	20	0	0	0	0	40
	Negative margins HPV negative	0	0	0	0	0	0	0
	Overall	40	51	11	7	9	2	120
mRNA-negative women subjet to follow-up	Positive margins HPV negative	0	3	0	0	0	0	3
	Negative margins HPV positive	2	0	0	0	0	0	2

**Table 3:** Residual disease (RD) according to mRNA results in study population.

	n	mRNA+	RD+	RD−	mRNA−	RD+	RD−
Positive margins	174						
Positive HPV	102	75 (73.5%)	65 (86.7%)	10 (13.3%)	27 (26.5%)	0	27 (100%)
Negative HPV	72	15 (20.9%)	15 (100%)	0 (0.0%)	57 (79.1%)	3 (5.3%)	54 (94.7%)
Negative margins	111						
Positive HPV	111	50 (45%)	40 (80%)	10 (20%)	61 (55%)	2 (3.3%)	59 (96.7%)
Overall	285	140 (49.1%)	120 (85.7%)	20 (14−3%)	145 (50.9%)	5 (3.4%)	140 (96.6%)

**Table 4:** Odds ratio for residual disease (RD).

	n	RD+	RD−	OR	CI 95%	*p*
Positive margins	174	80	94	1.25	0.8–2.0	0.001
HPV persistence	213	110	103	4.06	2.2–7.6	0.001
Positive E6/E7 mRNA	140	120	20	168	61.2–461.2	0.001

**Table 5:** Sensitivity, specificity, PPV and NPV of positive margin, HPV test and mRNA test for residual disease.

	Sensitivity	Specificity	PPV	NPV
Positive margin	64% (95%CI 58.1–69.5)	41.3% (95%CI 35.5–47.2)	46% (95%CI 40–52)	59.5% (95%CI 53.5–65.2)
HPVpersistence	88% (95%CI 83.5–91.4),	35.6% (95%CI 30.1–41.5)	52% (95%CI 46–57.6)	79.2% (95%CI 74–93.6)
E6/E7 mRNA	86% (95%CI 81–90)	96.6% (95%CI 93.5–98.2)	96% (95%CI 93–98)	88% (95%CI 83–91)

## Data Availability

The data presented in this study are available within the article.
